# Incidence of fidaxomicin allergy in patients with macrolide allergies: a large database analysis

**DOI:** 10.1128/aac.01924-24

**Published:** 2025-03-04

**Authors:** Chia-Yu Chiu, Daniel B. Chastain, Madison E. Salam, Joseph Sassine, Andrés F. Henao-Martínez

**Affiliations:** 1 Department of Medicine, Division of Infectious Diseases, University of Colorado 209664 , Aurora, Colorado, USA; 2 Department of Clinical and Administrative Pharmacy, University of Georgia College of Pharmacy 1355 , Albany, Georgia, USA; 3 Department of Pharmacy, University of Colorado Hospital, Aurora, Colorado, USA; 4 Department of Medicine, Infectious Diseases Section, The University of Oklahoma Health Sciences Center 6186 , Oklahoma City, Oklahoma, USA; University of Pittsburgh School of Medicine, Pittsburgh, Pennsylvania, USA

**Keywords:** azithromycin, clarithromycin, erythromycin, fidaxomicin, drug hypersensitivity

## Abstract

Fidaxomicin may exhibit cross-reactivity in patients with known macrolide allergies. In this analysis, compared to patients without macrolide allergies, the odds of fidaxomicin allergy were 2.31, 8.37, and 1.58 times higher in patients with azithromycin, clarithromycin, and erythromycin allergies, respectively; the absolute risk of fidaxomicin allergy was 0.033, 0.01, and 0.039 in patients with azithromycin, clarithromycin, and erythromycin allergies, respectively. The highest risk of anaphylaxis and angioedema was observed within 1 year of a non-fidaxomicin macrolide allergy.

## INTRODUCTION

Adverse event rates for non-fidaxomicin macrolides range from 6% to 27%, with hypersensitivity reactions occurring in only 0.4% to 3% of cases (1, 2). Due to structural similarities, the fidaxomicin package insert warns clinicians about the potential for cross-reactivity in patients with known macrolide allergies (3). We conducted a retrospective analysis using the TriNetX database to investigate the incidence of patients with pre-existing non-fidaxomicin macrolide allergies who experienced allergic reactions to fidaxomicin.

On 1 September 2024, we queried TriNetX (4), a global research network database that includes data from 100 million patients of all ages across more than 80 medical centers in multiple countries. The supplemental methods provide detailed information on the TriNetX framework and study design. Our group has published several reports using the same methodology, including studies on adverse drug reactions (5, 6).

### Baseline characteristic macrolide allergy

At the time of analysis, 129,605,248 patients were included in the TriNetX database. We identified 147,983, 12,653, 31,683, and 1,902 patients with documented allergies to azithromycin, clarithromycin, erythromycin, and fidaxomicin, respectively. The estimated incidence rates of allergy to azithromycin, clarithromycin, erythromycin, and fidaxomicin were 114.2 (95% CI 113.6–114.8), 9.8 (95% CI 9.6–9.9), 24.5 (95% CI 24.2–24.7), and 1.47 (95% CI 1.40–1.54) per 100,000 patients, respectively.

**Fig 1:**
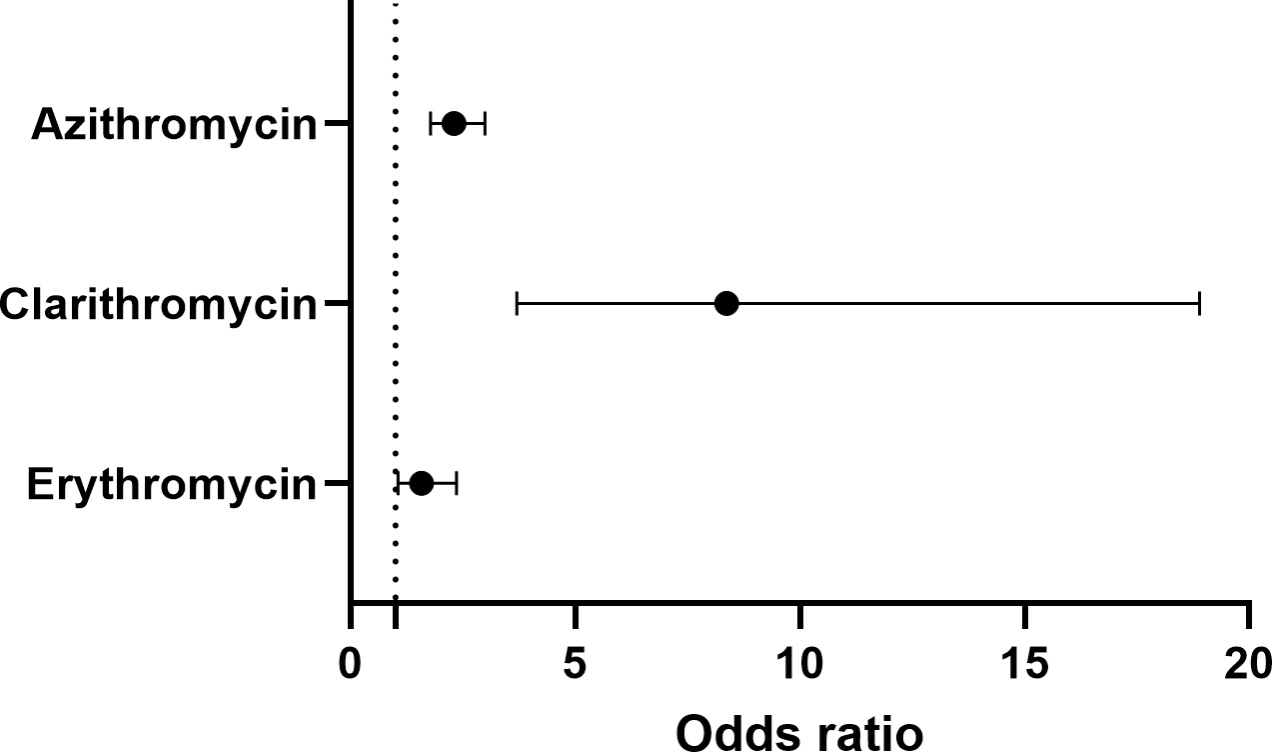
Odds ratio of fidaxomicin allergy in patients with other macrolide allergy. The odds ratios of fidaxomicin allergic reactions in patients with a history of azithromycin, clarithromycin, and erythromycin allergy were 2.31 (95% CI 1.78–2.99), 8.37 (95% CI 3.70–18.90), and 1.58 (95% CI 1.06–2.36), respectively.

Compared to patients without macrolide allergies, the odds of an allergic reaction to fidaxomicin were 2.31 times (95% CI 1.78–2.99) higher in patients with an azithromycin allergy, 8.37 times (95% CI 3.70–18.90) higher with a clarithromycin allergy, and 1.58 times (95% CI 1.06–2.36) higher with an erythromycin allergy (Fig. 1). The absolute risk of an allergic reaction to fidaxomicin was 0.033 in patients with an azithromycin allergy (80 of the 2,410 patients with a documented azithromycin allergy developed fidaxomicin allergy), 0.01 in patients with a clarithromycin allergy (10 of the 997 patients with a documented clarithromycin allergy developed fidaxomicin allergy), and 0.039 in patients with an erythromycin allergy (44 of the 1,124 patients with a documented erythromycin allergy developed fidaxomicin allergy) (Table S2).

### Allergy to non-fidaxomicin macrolide and fidaxomicin

**Fig 2:**
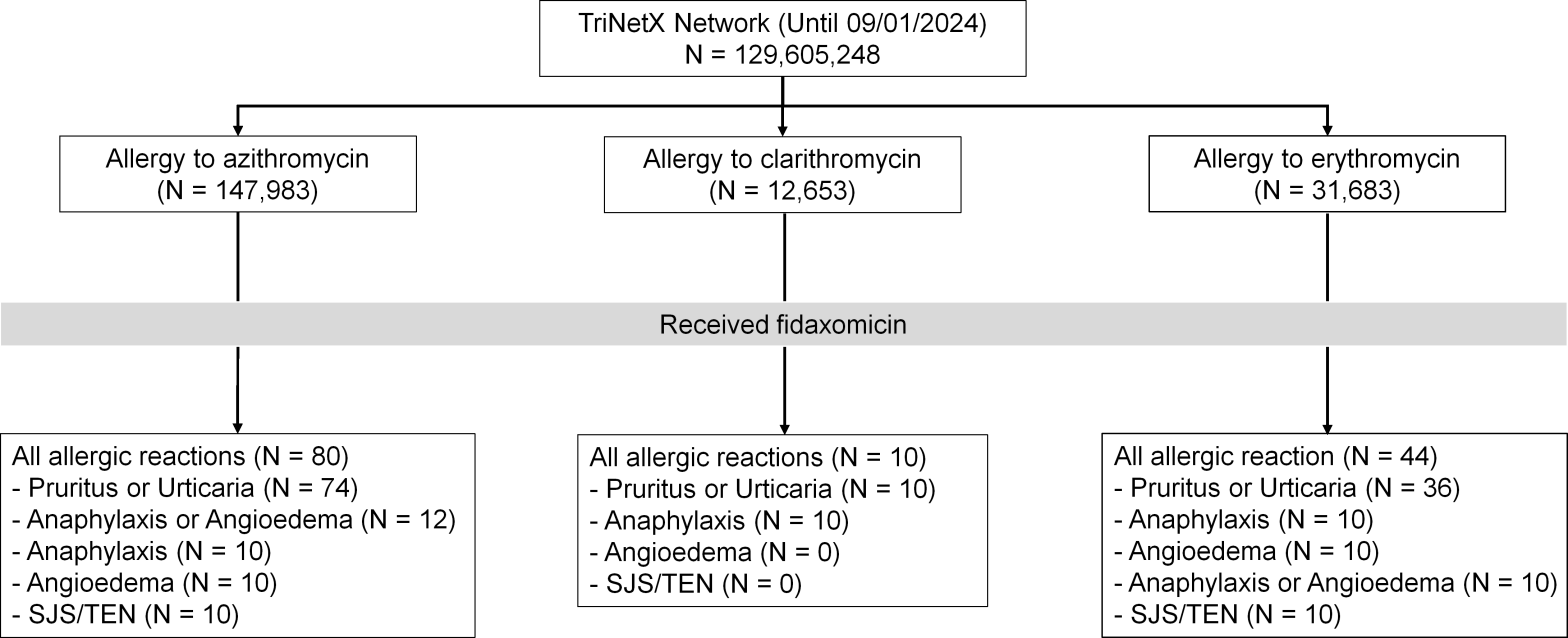
Patients with a non-fidaxomicin macrolide allergy label experienced allergic reactions to fidaxomicin. SJS/TEN, Stevens-Johnson syndrome/toxic epidermal necrolysis.

In total, 80 patients with a documented azithromycin allergy, 10 with a clarithromycin allergy, and 44 with an erythromycin allergy developed an allergic reaction to fidaxomicin (Table S3; Fig. 2). Anaphylaxis/angioedema and SJS/TEN were more frequent in patients whose azithromycin allergy was recorded within 1 year compared to those documented more than 1 year earlier (83% vs. 17% and 100% vs. 0%, respectively). All 10 patients with a clarithromycin allergy experienced anaphylaxis and pruritus/urticaria, with no cases of angioedema or SJS/TEN. In addition, these patients were all labeled with clarithromycin allergies within 1 year before fidaxomicin administration. Anaphylaxis/angioedema occurred exclusively in patients with erythromycin allergies documented within 1 year before fidaxomicin administration. By contrast, SJS/TEN occurred exclusively in patients labeled with erythromycin allergy more than 1 year before fidaxomicin administration. Notably, patients with anaphylaxis/angioedema were distinct from those with SJS/TEN across all groups (Table 1).

**TABLE 1:** Patients with non-fidaxomicin macrolide allergies documented within 1 year or more than 1 year before experiencing allergic reactions to fidaxomicin^*a*^

	Total	≤1 year	>1 year
Azithromycin			
All allergic reactions	80	43 (54%)	37 (46%)
Pruritus or urticaria	74	42 (57%)	32 (43%)
Anaphylaxis or angioedema^*b*^	12	10 (83%)	2 (17%)
SJS/TEN^*b*^	10	10 (100%)	0 (0%)
Clarithromycin			
All allergic reactions^*c*^	10	10 (100%)	0 (0%)
Erythromycin			
All allergic reactions	44	26 (59%)	18 (41%)
Pruritus or urticaria	36	23 (64%)	13 (36%)
Anaphylaxis or angioedema^*b*^	10	10 (100%)	0 (0%)
SJS/TEN^*b*^	10	0 (0%)	10 (100%)

^*a*^ SJS/TEN, Stevens-Johnson syndrome/toxic epidermal necrolysis.

^*b*^ The patients with anaphylaxis/angioedema were distinct from those with STS/TEN.

^*c*^ All of these 10 patients had anaphylaxis, pruritus, and urticaria symptoms.

Fidaxomicin contains an 18-membered lactone ring and has negligible systemic absorption, with plasma concentrations below 20 ng/mL, primarily remaining with the colon (2, 7). By contrast, macrolides with a 14-membered lactone ring (clarithromycin, erythromycin) or a 15-membered lactone ring (azithromycin) have better oral bioavailability (2, 7, 8). Due to their structural similarities, these macrolides may theoretically exhibit cross-reactivity (2, 3). However, this may not occur frequently because of the negligible systemic absorption of fidaxomicin. In addition, hypersensitivity reactions and allergies to macrolides are relatively uncommon compared to other antibiotics (2, 9), making it challenging to study cross-reactions between different macrolides.

In clinical trials, no cases of anaphylaxis due to fidaxomicin have been reported, or patients with a known fidaxomicin allergy were excluded from participation (10–12). The most common allergic reactions to fidaxomicin were gastrointestinal symptoms (5–10%), neutropenia (2%), and anemia (2%) (3). In post-marketing data, two small case series reported a relationship between fidaxomicin allergies and prior allergies to other macrolides (Table S4) (8, 13). Iarikov et al. reported three females (two with erythromycin allergy and one with azithromycin/erythromycin allergy) developed hypersensitivity reactions (one with pruritus/urticaria, one with throat itching without angioedema, and one with angioedema) after receiving fidaxomicin (13). Conversely, Kufel et al. reported no case of fidaxomicin allergy or intolerance in 11 patients with a history of macrolide allergy (eight with erythromycin allergy and three with azithromycin allergy) (8). In our study, anaphylaxis/angioedema predominantly occurred within 1 year of a non-fidaxomicin macrolide allergy. However, in patients with a clarithromycin allergy, all experienced anaphylaxis alone without angioedema (Table 1). Importantly, we observed that a non-fidaxomicin macrolide allergy increases the risk of fidaxomicin allergy (Fig. 1).

Cutaneous manifestations of antibiotic allergies encompass a broad spectrum of clinical phenotypes with varying onset times and immune mechanisms (14). In our study, pruritus/urticaria were the most common allergic reactions but were not reported in patients with a history of clarithromycin allergy more than 1 year before fidaxomicin administration. In addition, SJS/TEN occurred exclusively in patients with an azithromycin allergy documented within 1 year before fidaxomicin administration. In comparison, it occurred exclusively in those with an erythromycin allergy documented more than 1 year before fidaxomicin administration (Table 1). Although drug eruptions or pruritus occurred in less than 2% of patients receiving fidaxomicin in *Clostridioides difficile* infection (CDI) clinical trials (3), a handful of hypersensitivity cases have been reported post-marketing in the FDA adverse Event Reporting System (15). It is possible that in patients with non-fidaxomicin macrolide allergies, re-exposure to a similar chemical structure, even with minimal serum levels of fidaxomicin, could trigger severe hypersensitivity or severe cutaneous adverse events.

Our study has several limitations, primarily due to its retrospective design and the available information in TriNetX. Second, the reported macrolide allergies were presumably self-reported, and we could not confirm the index reactions. Cross-reactivity would ostensibly produce the same reaction, mediated by the same immune pathway (16). Therefore, dissimilar reactions may indicate a lower likelihood of true cross-reactivity. Literature on penicillin allergies indicates that self-reported allergies are less likely to involve cross-reactivity to cefazolin, underscoring the importance of validating and characterizing patients’ allergies (17). Third, fidaxomicin intolerance was not included in our search because these gastrointestinal symptoms are difficult to differentiate from those of CDI.

In summary, although macrolide allergies are infrequent, a history of non-fidaxomicin macrolide allergy slightly increases the risk of fidaxomicin allergy, particularly within 1 year of the initial allergy. Given the low absolute risk of fidaxomicin allergic reactions in patients with macrolide allergies and the minimal systemic absorption of fidaxomicin, we recommend accurately documenting the history of non-fidaxomicin macrolide allergies and closely monitoring patients for adverse drug reactions when administering fidaxomicin.

## SUPPLEMENTAL MATERIAL

Supplemental material aac.01924-24-s0001.docx Supplemental methods and Tables S1 to S4.

ASM does not own the copyrights to Supplemental Material that may be linked to, or accessed through, an article. The authors have granted ASM a non-exclusive, world-wide license to publish the Supplemental Material files. Please contact the corresponding author directly for reuse.
