# Supplementary material for: Incidence of fidaxomicin allergy in patients with macrolide allergies: a large database analysis
Source: Antimicrob Agents Chemother. 2025 Mar 4;69(4):e01924-24. doi: 10.1128/aac.01924-24 (PMC11963595; doi:10.1128/aac.01924-24)
Supplement: Supplemental material — Supplemental methods and Tables S1 to S4. [file aac.01924-24-s0001.docx]

**Supplementary Material**

**Supplementary Methods**

**Supplementary Table 1.** Diagnosis codes for the study population.

**Supplementary Table 2.** Fidaxomicin allergy events in patients with and without a history of azithromycin, clarithromycin, and erythromycin allergies.

**Supplementary Table 3.** Characteristics of patients with fidaxomicin allergy who had documented azithromycin, clarithromycin, and erythromycin allergies.

**Supplementary Table 4.** Case series of patients who developed fidaxomicin allergies and had a history of non-fidaxomicin macrolide allergies.

**Supplementary Methods**

Global Federated Research Network

TriNetX aggregates anonymized data from approximately 100 million patients of all ages across more than 80 medical centers in the US, Canada, Europe, Australia, Indonesia, and other countries. Each participating healthcare organization (HCO) contributes data from electronic medical record (EMR) systems used for patient care. Received data are either structured (e.g., coded diagnoses) or unstructured (e.g., provider notes) and processed by Natural Language Processing (NLP) Technology. Most participating HCOs are large academic medical institutions with both inpatient and outpatient facilities. The data provided represents the entire patient population at each HCO. Most give an average of seven years of historical data.

TriNetX receives data directly from each HCO's research repository in the TriNetX environment, or the HCO sends comma-separated value (CSV) files coded in the TriNetX Data Dictionary. Data providers update their information regularly, with over 80 percent of participants refreshing every one-, two-, or four weeks. The average lag time for an HCO's source data to refresh is one month.

TriNetX maps the data to a standard, controlled set of clinical terminologies and enters clinical information into a proprietary data model. The model entry includes extensive data quality assessment, and any records failing to meet the TriNetX quality standards are rejected. TriNetX is certified by the International Organization for Standardization (ISO) 27001:2013 and maintains an Information Security Management System (ISMS) to protect patient healthcare data and satisfies the requirements of the HIPAA Security Rule. Individualized patient data were obtained directly from the TriNetX platform in several CSV files. For patients with multiple encounters, we ensured the appropriate counting of distinct events.

Ethics Statement

Any data displayed on the TriNetX platform in aggregate form, or any patient-level data provided in a data set generated by the TriNetX platform, only contains de-identified data as per the de-identification standard defined in Section 164.514(a) of the Health Insurance Portability and Accountability Act (HIPAA) Privacy Rule. The process of de-identifying data is attested to through a formal determination by a qualified expert as defined in Section 164.514(b)(1) of the HIPAA Privacy Rule. Geographic reporting at the regional level prevents potential re-identification through the localization of patients or HCOs. Research utilizing TriNetX does not require ethical approval because patient-identifiable information is not accessible to users.

Study design and inclusion criteria

This analysis investigated the incidence and characteristics of patients with documented allergies to non-fidaxomicin macrolides (azithromycin, clarithromycin, and erythromycin) who subsequently experienced allergic reactions to fidaxomicin. Antibiotics were captured through RxNorm codes. History of non-fidaxomicin macrolide allergy reaction was identified using the International Classification of Diseases, Tenth Revision, Clinical Modification (ICD-10-CM) codes. Adverse events to fidaxomicin and its allergic reactions were identified using the ICD-10-CM codes, including anaphylactic shock/angioedema (within 1 day of receiving fidaxomicin), pruritus/urticaria (within 7 days of receiving fidaxomicin), Stevens-Johnson syndrome/Toxic epidermal necrolysis (SJS/TEN) (within 1 month of receiving fidaxomicin) **(Supplementary Table 1)**. These timeframes between the first fidaxomicin administration and allergic reaction occurrence were determined based on a literature review (1). We also analyzed the time interval between the documented non-fidaxomicin macrolide allergy and the onset of fidaxomicin-related allergic reactions, categorizing patients as having non-fidaxomicin allergies within 1 year or more than 1 year prior to the fidaxomicin reaction.

Exclusion criteria

Patients with non-macrolide antibiotic allergies documented on the same day as fidaxomicin allergies were excluded because it is difficult to determine whether the allergic reaction was due to fidaxomicin or non-macrolide antibiotic, the latter being the more likely culprit of allergic reactions (1). Allergies related to non-macrolide antibiotics were identified using ICD-10-CM codes. Patients who had both septic and anaphylactic shock simultaneously were excluded **(Supplementary Table 1)**.

Outcome Measures

The primary outcome was the estimated incidence and characteristics of fidaxomicin allergy in patients with documented allergies to non-fidaxomicin macrolides. The secondary outcome was the calculated odds ratio of fidaxomicin allergy in these patients.

Statistical Analysis

Descriptive statistics were presented using medians for continuous variables and frequency distributions with proportions for categorical variables. The estimated incidence and odds ratio were calculated by MadCalc (version 23.0.2). The figure was produced using GraphPad Prism version 10.4.1.

**Reference**

1. Blumenthal KG, Peter JG, Trubiano JA, Phillips EJ. 2019. Antibiotic allergy. The Lancet 393:183–198.

2. Iarikov DE, Alexander J, Nambiar S. 2014. Hypersensitivity Reactions Associated With Fidaxomicin Use. Clinical Infectious Diseases 58:537–539.

3. Kufel WD, Hitchcock AM, Delbalso AN, Paolino KM. 2023. Evaluation of fidaxomicin use in patients with a macrolide allergy/intolerance. Journal of Antimicrobial Chemotherapy 78:1127–1128.

**Supplementary Table 1.** Diagnosis codes for the study population.

| **Allergy to Macrolides** | **ICD-10-CM** |
| --- | --- |
| Adverse event of Macrolides | T36.3 |
| Allergy to antibiotic agents | Z88.1 |
| **Allergy to non-Macrolide antibiotic** |  |
| Allergy or adverse event to penicillin | Z88.0, T36.0 |
| Allergy to sulfonamides | Z88.2 |
| Adverse event to cephalosporins or other beta-lactam antibiotics | T36.1 |
| Adverse event to tetracycline | T36.4 |
| Adverse event to aminoglycoside | T36.5 |
| Adverse event to rifampicin | T36.6 |
| Adverse event to systemic antifungal antibiotics | T36.7 |
| **Antibiotic** | **RxNorm Code** |
| Azithromycin | 18631 |
| Clarithromycin | 21212 |
| Erythromycin | 4053 |
| Fidaxomicin | 1111103 |
| **Symptoms and signs of allergy reaction** | **ICD-10-CM** |
| Anaphylactic shock | T78.2 |
| Angioedema | T78.3 |
| Pruritus | L29 |
| Stevens-Johnson syndrome / Toxic epidermal necrolysis | L15.1, L15.2, L15.3 |
| Urticaria | L50 |
| **Exclusion of other types of sepsis** | **ICD-10-CM** |
| Bacterial sepsis | A41.9 |

**Supplementary Table 2.** Fidaxomicin allergy events in patients with and without a history of azithromycin, clarithromycin, and erythromycin allergies.

|  | **Allergic reaction to fidaxomicin** | **No allergic reaction to fidaxomicin** |  |
| --- | --- | --- | --- |
| **History of azithromycin allergy** | 80 (A) | 2,330 (B) | 2,410 |
| **No history of azithromycin allergy** | 215 (C) | 14,454 (D) | 14,669 |
|  | 295 | 16,784 | 17,079 |

|  | **Allergic reaction to fidaxomicin** | **No allergic reaction to fidaxomicin** |  |
| --- | --- | --- | --- |
| **History of clarithromycin allergy** | 10 (A) | 987 (B) | 997 |
| **No history of clarithromycin allergy** | 14 (C) | 11,569 (D) | 11,583 |
|  | 24 | 12,556 | 12,580 |

|  | **Allergic reaction to fidaxomicin** | **No allergic reaction to fidaxomicin** |  |
| --- | --- | --- | --- |
| **History of erythromycin allergy** | 44 (A) | 1,080 (B) | 1,124 |
| **No history of erythromycin allergy** | 59 (C) | 2,293 (D) | 2,352 |
|  | 103 | 3,373 | 3,476 |

Footnote: Absolute risk = A / A+B; Odds ratio = A x D / B x C

**Supplementary Table 3.** Characteristics of patients with fidaxomicin allergy who had documented azithromycin, clarithromycin, and erythromycin allergies.

|  | **Azithromycin**  **N = 80** | **Clarithromycin ^a^**  **N = 10** | **Erythromycin**  **N = 44** |
| --- | --- | --- | --- |
| **Median Age** | 56 | - | 55 |
| **Age < 18** | 2 (3) |  | 3 (7) |
| **Gender** |  |  |  |
| Male | 16 (20) | - | 9 (20) |
| Female | 64 (80) | - | 35 (80) |
| Unknown | 0 (0) | - | 0 (0) |
| **Ethnicity** |  |  |  |
| Hispanic | 6 (8) | - | 10 (23) |
| Non-Hispanic | 67 (84) | - | 34 (77) |
| Unknown | 7 (9) | - | 0 (0) |
| **Race** |  |  |  |
| White | 62 (78) | - | 30 (68) |
| Black | 8 (10) | - | 7 (16) |
| Asian | 5 (6) | - | 7 (16) |
| Unknown or Other | 5 (6) | - | 0 (0) |

1. The number of patients in this category is too small. TriNetX did not display detailed information to ensure data deidentification.

**Supplementary Table 4.** Case series of patients who developed fidaxomicin allergies and had a history of non-fidaxomicin macrolide allergies.

| **Author,**  **Country,**  **reference** | **Study design** | **Patient characteristic** |
| --- | --- | --- |
| Iarikov et al.,  USA,  (2) | Retrospective,  FDA Adverse Event Reporting System until 2014 | Among 12 cases with fidaxomicin-related hypersensitivity, 3 had a history of macrolide allergy. Fidaxomicin was discontinued in all 3 cases.  Case 1: 49F. Azithromycin/erythromycin allergy. The patient developed a diffused rash and itching after the first dose of fidaxomicin.  Case 2: 79F. Erythromycin allergy. The patient developed a burning sensation in the throat after 2 doses of fidaxomicin.  Case 3: unknown age/female. Erythromycin allergy. The patient developed swelling of the throat and chest after 2 days of fidaxomicin. |
| Kufel et al.,  USA,  (3) | Single center retrospective study,  1/1/2013-1/11/2022 | Eleven patients with macrolide-related allergy or intolerance (8 erythromycin, 3 azithromycin). All received fidaxomicin 200 mg BID for 10 days, and none reported adverse reactions related to fidaxomicin. |
